# Antimicrobial, Antioxidant, Anti-Acetylcholinesterase, Antidiabetic, and Pharmacokinetic Properties of *Carum carvi* L. and *Coriandrum sativum* L. Essential Oils Alone and in Combination

**DOI:** 10.3390/molecules26123625

**Published:** 2021-06-13

**Authors:** Hafedh Hajlaoui, Soumaya Arraouadi, Emira Noumi, Kaïss Aouadi, Mohd Adnan, Mushtaq Ahmad Khan, Adel Kadri, Mejdi Snoussi

**Affiliations:** 1 Research Unit Valorization and Optimization of Resource Exploitation (UR16ES04), Faculty of Science and Technology of Sidi Bouzid, University of Kairouan, Campus University Agricultural City, Sidi Bouzid 9100, Tunisia; bio.hafedh@gmail.com; 2 Regional Center of Agricultural Research (CRRA) Sidi Bouzid, Gafsa Road Km 6, PB 357, Sidi Bouzid 9100, Tunisia; bio.soumaya@gmail.com; 3 National Research Institute for Rural Engineering, Water and Forestry (INRGREF), University of Carthage, 10 Street Hédi Karray, Manzeh IV, Ariana 2080, Tunisia; 4 Department of Biology, College of Science, Hail University, P.O. Box 2440, Ha’il 81451, Saudi Arabia; emira_noumi@yahoo.fr (E.N.); drmohdadnan@gmail.com (M.A.); snmejdi@yahoo.fr (M.S.); 5 Laboratory of Bioressources—Integrative Biology & Recovery, High Institute of Biotechnology, University of Monastir, Monastir 5000, Tunisia; 6 Department of Chemistry, College of Science, Qassim University, Buraidah 51452, Saudi Arabia; kaiss_aouadi@hotmail.com; 7 Faculty of Sciences of Monastir, Avenue of the Environment, University of Monastir, Monastir 5019, Tunisia; 8 Department of Microbiology and Immunology, College of Medicine and Health Sciences, UAE University, Al Ain 17666, United Arab Emirates; 9 Faculty of Science and Arts in Baljurashi, Albaha University, P.O. Box 1988, Albaha 65731, Saudi Arabia; lukadel@yahoo.fr; 10 Department of Chemistry, Faculty of Science of Sfax, University of Sfax, B.P. 1171, Sfax 3000, Tunisia; 11 Laboratory of Genetic, Biodiversity and Valorization of Bioressources, Higher Institute of Biotechnology of Monastir, University of Monastir, Avenue Taher Hadded BP 74, Monastir 5000, Tunisia

**Keywords:** *Carum carvi*, *Coriandrum sativum*, essential oil, antioxidant, antimicrobial, anti-acetylcholinesterase, antidiabetic, pharmacokinetics

## Abstract

Herbs and spices have been used since antiquity for their nutritional and health properties, as well as in traditional remedies for the prevention and treatment of many diseases. Therefore, this study aims to perform a chemical analysis of both essential oils (EOs) from the seeds of *Carum carvi* (*C. carvi*) and *Coriandrum sativum* (*C. sativum*) and evaluate their antioxidant, antimicrobial, anti-acetylcholinesterase, and antidiabetic activities alone and in combination. Results showed that the EOs mainly constitute monoterpenes with γ-terpinene (31.03%), β-pinene (18.77%), p-cymene (17.16%), and carvone (12.20%) being the major components present in *C. carvi* EO and linalool (76.41%), γ-terpinene (5.35%), and α-pinene (4.44%) in *C. sativum* EO. In comparison to standards, statistical analysis revealed that *C. carvi* EO showed high and significantly different (*p* < 0.05) antioxidant activity than *C. sativum* EO, but lower than the mixture. Moreover, the mixture exhibited two-times greater ferric ion reducing antioxidant power (FRAP) (IC_50_ = 11.33 ± 1.53 mg/mL) and equipotent chelating power (IC_50_ = 31.33 ± 0.47 mg/mL) than the corresponding references, and also potent activity against 2,2-diphenyl-1-picrylhydrazyl (DPPH) (IC_50_ = 19.00 ± 1.00 mg/mL), β-carotene (IC_50_ = 11.16 ± 0.84 mg/mL), and superoxide anion (IC_50_ = 10.33 ± 0.58 mg/mL) assays. Antimicrobial data revealed that single and mixture EOs were active against a panel of pathogenic microorganisms, and the mixture had the ability to kill more bacterial strains than each EO alone. Additionally, the anti-acetylcholinesterase and α-glucosidase inhibitory effect have been studied for the first time, highlighting the high inhibition effect of AChE by *C. carvi* (IC_50_ = 0.82 ± 0.05 mg/mL), and especially by *C. sativum* (IC_50_ = 0.68 ± 0.03 mg/mL), as well as the mixture (IC_50_ = 0.63 ± 0.02 mg/mL) compared to the reference drug, which are insignificantly different (*p* > 0.05). A high and equipotent antidiabetic activity was observed for the mixture (IC_50_ = 0.75 ± 0.15 mg/mL) when compared to the standard drug, acarbose, which is about nine times higher than each EO alone. Furthermore, pharmacokinetic analysis provides some useful insights into designing new drugs with favorable drug likeness and safety profiles based on a *C. carvi* and *C. sativum* EO mixture. In summary, the results of this study revealed that the combination of these EOs may be recommended for further food, therapeutic, and pharmaceutical applications, and can be utilized as medicine to inhibit several diseases.

## 1. Introduction

Plant essential oils (EOs) and their bioactive components extracted from herbs and spices have received increasing interest as new natural alternatives in many different areas such as the pharmaceutical, cosmetic, and food industries [1,2]. Their apparently safe nature, along with their potential effectiveness and well-acknowledged antioxidant capacity, help to provide remedies to mankind against various diseases [3,4]. The exposure of the human body to different types of reactive species such as free radicals (ROS/RNS) induces oxidative stress that leads to lipid peroxidation, protein glycation/oxidation and nitration, enzyme inactivation, and DNA damage, which leads to the development of various pathological conditions such as diabetes mellitus (DM) and neurodegenerative diseases, which in turn can be neutralized by the presence of endogenous or exogenous antioxidant systems [5,6,7]. In addition, emerging infectious and chronic diseases due to rising antibiotic resistance are responsible for several epidemics and pandemics with disastrous consequences, and therefore pose a serious threat to public health [8,9]. Along with the drastically increased interest from consumers in the use of natural agents to treat a variety of ailments, there have been numerous reports that have described the beneficial effects of using EOs from culinary herbs that are usually considered a crucial pillar of healthcare with high safety and no side effects [10]. EOs and their molecules can modulate different signaling pathways that are over activated or down regulated during acute or chronic inflammatory responses [11].

Coriander (*Coriandrum sativum* L.) is an herbaceous and glabrous medicinal and aromatic plant, belonging to the family of *Umbelliferae* (*Apiaceae*), typically found in several parts of the world such as the Mediterranean and Middle Eastern regions, with India being the biggest producer, consumer, and exporter [12]. This herb is widely used in the Mediterranean region for the treatment of gastrointestinal disorders such as anorexia and diarrhea, as well as to alleviate spasms, gastric complaints, bronchitis, and gout [13]. Tunisian people widely use this condiment as a flavoring and adjuvant agent and/or as traditional remedies for the treatment of different diseases such as insufficient milk, postpartum, and eczema. It has been reported that coriander possess a broad spectrum of therapeutic effects, including antioxidant, antimutagenic, antidiabetic, sedativehypnotic, antihelmintic, anticonvulsant, diuretic, antifungal, anxiolytic, anticancer, hepatoprotective, etc. [14]. The EO of coriander is composed of many bioactive molecules mainly used in the food and cosmetic industries, because of its perfume and antioxidant potential [15]. Caraway (*Carum carvi* L.) is an aromatic, biennial umbelliferous plant belonging to the *Apiaceae* family. This plant has been served traditionally for a long time for various medical prescriptions such as digestive unrest including gas, loss of appetite, bloating, heartburn, as well as mild spasms of the stomach and intestines [16]. Traditionally, caraway oil has been used to help people to avoid phlegm, relieve constipation, and provide urination control. Nursing mothers use caraway to increase the flow of breast milk [17]. Many pharmacological potencies of caraway seeds have been demonstrated such as anti-convulsant, antimicrobial, analgesic, anti-inflammatory, anti-anxiety, anti-hyperglycemic, and anti-spasmodic properties, and it has been used as a cure for dyspepsia, flatulent indigestion, diarrhea, and hysteria [18]. Caraway fruits are used in various traditional systems as curative plants for the management of different ailments, such as digestive disorders, functional dyspepsia, thyroid hormones, as well as a remedy to cure indigestion, pneumonia, carminative, appetizer, and galactagogue [19,20,21]. Also, it has been used as an important ingredient in anti-obesity drugs in Unani traditional medicine [22], largely employed to reduce the plasma triglycerides and cholesterol levels in normal and streptozotocin rats [23].

These two spices have been widely exploited as remedies in traditional folk medicine for many diseases globally. A combination of their EOs is a novel alternative to create better medication and create a synergistic blend creating even more benefits. 

In continuation of our research to discover potential natural therapeutic agents [24,25,26,27,28,29,30,31,32,33], this study aimed to investigate the composition of *C. carvi* and *C. sativum* EOs by GC-MS analysis. To elucidate their pharmacotherapeutic virtues, we assessed their antioxidant and antimicrobial activities, and we reported for the first time their anti-acetylcholinesterase and antidiabetic inhibitory effect alone and in combination. As a part of our endeavor to increase the potential and exploration of these activities, a pharmacokinetics study has been performed.

## 2. Results

### 2.1. Chemical Composition of C. carvi and C. sativum EOs

In this study, the *C. carvi* and *C. sativum* EOs prepared by the hydro-distillation method have a yield of 1.32% and 0.83% (*v*/*w*) based on dry weight, respectively. EOs were characterized by gas chromatography coupled with flame ionization detectors (GC-FID) and mass spectrometry (GC-MS), resulting in the identification of 28 volatile compounds for *C. carvi* EO, representing 99.1% of the total oil composition, with the major compounds being γ-terpinene (31.03%), β-pinene (18.77%), *p*-cymene (17.16%), and carvone (12.20%). Meanwhile, *C. sativum* EO highlighted 27 components representing 99.46% with high levels of linalool (76.41%), followed respectively by γ-terpinene (5.35%) and α-pinene (4.44%) (Table 1). Our results were different to what has been previously reported in other provenances of *C. carvi* EO, demonstrating that it was essentially dominated by γ-carvone (45–95%), followed by γ-limonene (1.5–51%) [19,34]. It has been reported that EOs from three Tunisian ecotypes were dominated by carvone (76.8–80.5%) and limonene (13.1–20.3%) [35]. Chinese caraway seed EO contain limonene (43.5%), carvone (32.6%), and apiole (15.1%) as major components [36], however only carvone (65.77 to 78.8%) and limonene (19.38 to 31.64%) are very abundant in other EOs [37]. EO cultivated from *C. carvi* in the high hills of the Uttarakhand Himalayas mainly contains carvone (44.5–95.9%), limonene (1.5–51.3%), β-myrcene (0–0.4%), trans-dihydrocarvone (0–0.5%), and trans-carveole (0–0.2%) [38]. Based on the results of the chemical composition of *C. carvi* EO from different ecotypes, it has been found that carvone (61.6–77.4%) and limonene (16.2–29.1%) were the major components of caraway oils. German caraway EO mainly contains carvone (77.3%) and limonene (16.2%), while the corresponding values were 76.3% and 19.5% for other Tunisian chemotypes. Carvone (61.6%), limonene (29.1%), β-myrcene (3.9%) and α-selinene (10.9%) were the main components of Egyptian chemotype [39]. 

The EO composition of *C. sativum* from the same organ has been widely studied and the literature survey showed a great variability in essential oil composition, where the main detected compounds were linalool (55.59%), γ-terpinene (7.47%), α-pinene (7.14%), and camphor (5.59%) [40], similar to that obtained in our study. Gil et al. [41] reported that the main components found in Argentinean and European coriander EO are linalool (72.3% and 77.7%), followed by α-pinene (5.9% and 4.4%), γ-terpinene (4.7% and 5.6%), camphor (4.6% and 2.4%), and limonene (2.0% and 0.9%, respectively). Also, linalool (65.8%), α-pinene (6.8%), γ-terpinene (6.1%), and camphor (5.1%), were found in the EO from New Zealand [42]. Similarly, Russian coriander seed EO was dominated by linalool (68.0%) [43]. Consequently, we can conclude the predominant presence of linalool in a large number of wild coriander seed EO all over the world.

**Table 1:** Chemical composition (%) of the EOs isolated from the seeds of two Tunisian spices *C. carvi* and *C. sativum*.

N^o^	Compounds Identified	RI ^a^	RI ^b^	(%) *C. carvi*	(%) *C. sativum*	Identification
1	α-thujene	921	927	0.34	tr	RI,MS
2	α-pinene	932	935	0.62	4.44	RI,MS,CS
3	Camphene	946	950	-	0.40	RI,MS
4	Sabinene	969	974	-	0.21	RI,MS
**5**	**β-pinene**	979	981	**18.77**	0.66	RI,MS
6	Myrcene	992	991	1.00	0.56	RI,MS
7	Octanal	1004	1002	-	tr	RI,MS
8	α-phellendrene	1002	1006	0.22	-	RI,MS
9	δ-3-carene	1011	1011	0.06	-	RI,MS
10	α-terpinene	1014	1018	0.16	tr	RI,MS,CS
**11**	***p*-cymene**	1027	1026	**17.16**	1.74	RI,MS
**12**	Limonene	1032	1034	0.42	1.23	RI,MS
**13**	**γ-terpinene**	10,599	1064	**31.03**	5.35	RI,MS,CS
14	Cis-linalool oxide	1072	1076	**-**	0.5	RI,MS
15	Octanol	1070	1085	**-**	0.30	RI,MS
16	**Linalool**	1095	1089	tr	76.41	RI,MS,CS
17	Cis-sabinene hydrate	1098	1099	0.25	-	RI,MS
18	Trans-allo-ocimene	1128	1141	tr	-	RI,MS
19	**Camphor**	1146	1151	-	2.20	RI,MS
20	Citronellal	1148	1153	-	0.49	RI,MS
21	p-mentha-1,5-dien-8-ol	1166	1158	tr	-	RI,MS
22	Trans-pinocarveol	1145	1161	0.19	-	RI,MS
23	Borneol	1169	1179	0.18	0.23	RI,MS
24	Terpinen-4-ol	1177	1193	-	0.23	RI,MS
25	α-terpeniol	1195	1204	-	.026	RI,MS
26	Cryptone	1193	1195	0.41	-	RI,MS
27	Myrtenal	1196	1204	tr	-	RI,MS
28	Decanal	1205	1227	0.12	-	RI,MS
29	Citronellol	1230	1243	-	0.58	RI,MS
**30**	**Carvone**	1242	1247	**12.20**	-	RI,MS
**31**	**Cuminaldehyde**	1247	1254	**-**	0.90	RI,MS
32	Geraniole	1265	1261	-	0.15	RI,MS,CS
**33**	**1-phenylbutanol**	-	1289	**3.29**	-	RI,MS
34	Decanol	1277	1287	-	0.10	RI,MS
35	Safrole	1285	1293	-	0.27	RI,MS
**36**	**Bornyl acetate**	1292	1298	**12.84**	-	RI,MS,CS
37	(E)-anethole	1282	1302	tr	-	RI,MS
38	Undecanal	1308	1305	-	tr	RI,MS
39	δ-elemene	1334	1330	tr	-	RI,MS
40	α-terpenyl acetate	1346	1350	-	0.14	RI,MS
**41**	**Geranyl acetate**	**1379**	**1382**	0.14	1.81	RI,MS
42	α-copaene	1385	1383	0.15	-	RI,MS
43	Trans-caryophyllene	1417	1425	0.10	-	RI,MS
44	γ-selinene	-	1437	tr	-	RI,MS
**Total identification**				**99.10**	**99.46**	

^a^ RI = literature retention indices on HP-5MS column, according to Adams, 2007 [44]; ^b^ RI = experimental retention index calculated against a C6–C28 *n*-alkanes mixture on the HP-5MS column; % = percentage peak area of essential oil components; - = not detected; tr = traces (<0.1%); MS = mass spectra; CS = co-injection with authentic standard.

### 2.2. Antioxidant Activity Evaluation

The antioxidant activity was evaluated following five assays: DPPH, superoxide anion, reducing power, chelating power, and β-carotenes. The results (Table 2) revealed that *C. carvi* EO (IC_50_ = 34.00 ± 3.46 mg/mL) and *C. sativum* EO (IC_50_ = 38.83 ± 0.70 mg/mL) displayed moderate DPPH scavenging activity and were significantly different (*p* < 0.05) when compared to the mixture EO (IC_50_ = 19.00 ± 1.00 mg/mL), which exhibited the strongest antioxidant activity but was about two times weaker than the reference drug. Superoxide anion and β-carotene assays followed the same trend as DPPH assays but with less effectiveness. In contrast, potent and significantly different (*p* < 0.05) antioxidant activity was recorded for the mixture (EC_50_ = 11.33 ± 1.53 mg/mL) in the FRAP test, which is two times higher than the tested standard (EC_50_ = 23.00 ± 1.00 mg/mL), showcasing the high effect of synergism between the EO components. However, *C. sativum* EO exhibited equipotent and insignificantly different (*p* > 0.05) reducing power to the control and was less potent than the *C. carvi* EO, but statistically different (*p* < 0.05). The chelating activity of *C. sativum* was found to be significantly different (*p* < 0.05) to *C. carvi*, mixture, and EDTA. Our results justified the finding that the addition of caraway and coriander to food increased the antioxidant content of the food due to the presence of antioxidant and anti-inflammatory compounds [45,46,47,48,49,50].

From the above data, the high antioxidant potential of *C. carvi* and *C. sativum* EOs and their mixture is due to their high terpene contents (Table 1). The non-phenolic compound, γ-terpinene, as a pre-aromatic monoterpene hydrocarbon, has demonstrated antioxidant activity and is also able to inhibit lipid peroxidation [51]. The addition of EO rich in γ-terpinene to edible lipids may provide an alternative or supplementary strategy for obtaining large increases in their oxidative stability and shelf life, as well as the addition of vitamin E [52]. Similar to previous studies, the high free radical scavenging ability of *C. sativum* may be ascribed also to the presence of carvone at a high level, because carvone has conjugated double bonding, and, hence, high antioxidant activity [53]. Moreover, carvone has been demonstrated as a protective agent against lipid peroxidation [54]. The alcohol linalool, which is the predominant component in *C. sativum* EO, plays an important role in the activity of EOs as a synergy component rather than a single antioxidant [53]. *p*-cymene, as a monoterpene, has been considered a high antioxidant activity constituent of complex natural mixtures [53]. The potent antioxidant power of *p*-cymene has been proven through its interactions with other monoterpenes, leading to synergistic interactions, which explains in part the strong antioxidant effect of the EO mixture in our study.

### 2.3. Antimicrobial Activity Evaluation

The results of antimicrobial potential are reported via the determination of IZ (inhibition zone), MIC, MBC, and MFC values of *C. carvi* and *C. sativum* EOs alone and in combination. Furthermore, antibiotics against eighteen human pathogenic strains were also used as standards in the present study, and are outlined in Table 3 and Table 4. Table 3 showed that IZDs are in the range of 11.00 ± 00 mm to 25.00 ± 0.00 mm for *C. carvi* EO, 8.33 ± 0.57 mm to 21.66 ± 1.15 mm for *C. sativum* EO, 12.00 ± 1.00 mm to 25.66 ± 0.57 mm for the mixture, and 17.00 ± 1.00 mm to 32.67 ± 0.58 mm for the standards (tetracycline and gentamycin). 

In addition, *C. carvi* exhibited potent antifungal activity (Table 4) when compared to *C. sativum* against all strains (except equipotent activity with *C. glabrata*), however the mixture displayed more potent activity as compared to *C. carvi* and *C. sativum* alone for all strains. The combined EOs displayed the same IZDs as the reference drug except for *C. parapsilosis* and *S. cerevisae* strains. IZDs, meaning a comparison of all tested microorganisms (bacteria and yeast), showed that the mixture has the highest activity (*p* < 0.05) compared to the two oils alone. Moreover, mixture IZDs for yeast strains were similar to amphotericin B except against *C. parapsilosis* and *S. cerevisiae.*

The results of MIC and MBC values against all strains ranged from 0.059 mg/mL (*M. luteus, E. feacalis*) to 1.875 mg/mL (*P. aeruginosa, V. natrigens*) and 0.234 mg/mL (*M. luteus*) to 3.750 mg/mL (*P. aeruginosa, V. parahaemolyticus, V. furnisii, V. mimicus, V. natrigens*) for *C. carvi* EO, and from 0.234 mg/mL (*S. aureus*, *M. luteus*, *V. parahaemolyticus*) to 1.875 mg/mL (*P. aeruginosa, V. alginolyticus, V. proteolyticus, V. furnisii, V. mimicus, V. natrigens, V. carhiaccae*) and 0.938 mg/mL (*S. aureus*, *M. luteus, E. feacalis*) to 7.500 mg/mL (*V. alginolyticus, V. furnisii, V. mimicus, V. carhiaccae*) for *C. sativum* EO. Interestingly, in the case of the EO mixture, MIC and MBC values have been reduced and were found to be in the range of 0.029 mg/mL (*S. epidermidis*) to 0.469 mg/mL (*P. aeruginosa, V. natrigens*) and 0.059 mg/mL (*M. luteus*) to 0.938 mg/mL (*P. aeruginosa, V. mimicus, V. natrigens*), which are very close to the used antibiotics.

To better underline the capability of EOs in destroying bacterial and fungal cells (bactericidal and fungicidal) or simple growth inhibition effects (bacteriostatic and fungistatic), the MBC/MIC and MFC/MIC ratios have been determined for each strain (Table 5 and Table 6). As shown, *C. carvi* EO was found to be bactericidal against all strains, except *E. faecalis*, *V. alginolyticus* and *V. furnisii*, and fungicidal against only *C. glabrata* and *C. parapsilosis*. Meanwhile, *C. sativum* was only bacteriostatic against *V. parahaemolyticus* and *V. fluvialis* and fungistatic against *C. krusei*. Additionally, the mixture was found to be more effective at killing some bacterial strains than each EO alone and even more so than standard drugs, especially against *B. cereus.*

**Figure 1:**
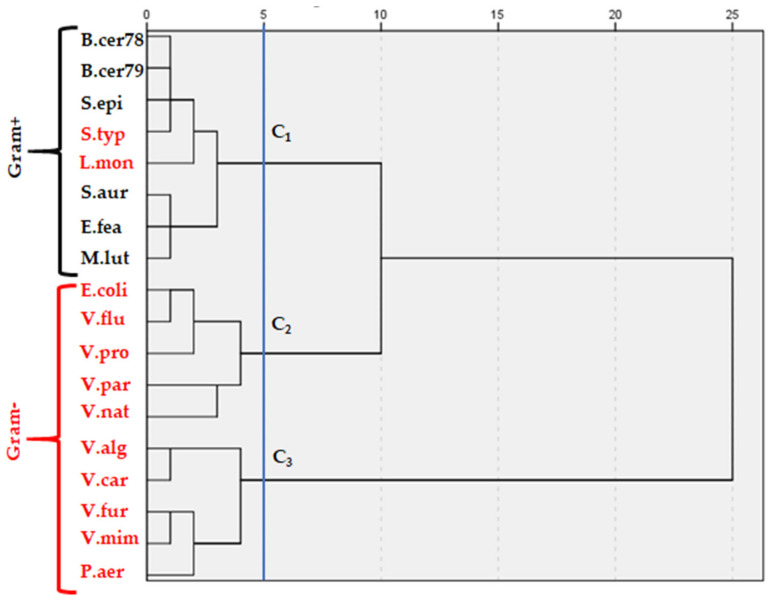
Hierarchical cluster analysis (HCA) of bacterial strains, according to their responses to all EOs, based on MIC and MBC values. Clustering was established according to ward method.

According to statistical analysis, means comparison of IZDs of all strains showed that Gram positive (Gram+) bacteria were more sensitive than Gram negative (Gram−). Moreover, the HCA (Figure 1) of these bacterial strains (Gram+ and Gram−), according to their responses to all EOs, based on the MIC and MBC values, allowed us to classify these stains on three clusters. The first one (C1) regroups all Gram+ bacteria, specifically *S. typhimurium* and *L. monocytogenes*, that reveal a similarity in their response against the assessed EO. Gram-strains, meanwhile, were clustered in two groups, C2 and C3, and according to the Ward clustering method C2 was found to be more related to C1 than C3.

As shown, the high resistance of Gram-negative bacteria when compared to the Gram-positive ones, may be due to their cell walls having a thick layer of peptidoglycan, making it difficult for antimicrobial agents to pass through, and thus imparting rigidity to their cells [55]. It has been mentioned previously that 75% of antibacterial drugs were terpenes [9]. The antimicrobial activity of EOs can vary with the type of the RO and the used microorganism. In this study, potent antimicrobial activity was especially observed for the mixture, mainly due to the richness of monoterpenes. Among them, carvone has been reported for its potential in inhibiting the growth of bacteria and fungi [56]. Also, carvone is known to possess potent inhibitory activity against *E. coli* and *S.* *typhimurium* [9]. Linaloole extracted from lavender EO by membrane disruption has demonstrated antimicrobial activity against resistant *K. pneumoniae* [57]. A previous study conducted by Giweli et al. [58] showed the strong antimicrobial activity of γ-terpinene that was obtained in high proportion in *C. carvi.*

### 2.4. Enzymes Inhibitory Activity Evaluation

#### 2.4.1. Cholinesterase Inhibition

Acetylcholinesterase ends the effect of this neurotransmitter at cholinergic synapses by hydrolyzing acetylcholine to choline and acetate. Therefore, the inhibition of cholinesterase enzymes (anti-AChE) is considered promising in the management of neurological and neurodegenerative disorders such as AD. In this study, we evaluated for the first time the anti-cholinesterase activity of EOs alone and in combination. Results (Table 7) showed that *C. carvi* (IC_50_ = 0.82 ± 0.05 mg/mL) and *C. sativum* (IC_50_ = 0.68 ± 0.03 mg/mL) EOs alone, as well as in combination (IC_50_ = 0.63 ± 0.02 mg/mL), significantly inhibited the acetylcholinesterase enzyme when compared to the reference drug galanthamine (IC_50_ = 1.05 ± 0.05 mg/mL). Statistical analysis indicates the similarly strong inhibition potency of the mixture and *C. sativum*, which are not significantly different (*p* > 0.05). However, the inhibitory effect of *C. carvi* was significantly different (*p* < 0.05) from those of *C. sativum* and the standard galanthamine. Our finding has been supported by Öztürk [59], who reported that EO rich in terpenes shows high AChE inhibitory activity, especially γ-terpinene (IC_50_ = 181 μg/mL). Tnidis et al. [60], in a similar work, reported the ability of terpenes from *Stachys lavandulifolia* Vahl (*Lamiaceae*) to inhibit AChE. Menichini et al. [61] indicated that the *Pimpinella anisoides* plant, which is rich in terpenes, exhibited enzyme inhibition towards AChE. The anti-AChE activity of *Salvia lavandulaefolia* Vahl EOs containing β-pinene and linalool, which are obtained in high proportion in our oils, have also been confirmed [62]. The study confirmed the potency of combined terpenes. In another study, linalool’s high AChE inhibition was confirmed [63].

#### 2.4.2. Antidiabetic Inhibition

T2DM is a metabolic disorder characterized by persistent hyperglycemia with serious complications. α-glucosidase is one of the enzymes involved in the deconstruction of long chain carbohydrates by breaking down starch and disaccharides to glucose. To reduce the high levels of glucose in the blood, enzyme inhibitors are a key. An anti-diabetic bioassay was carried out for the first time to test *C. carvi, C. sativum*, and a mixture of both EOs as α-glucosidase inhibitors. It was obvious that *C. carvi* (IC_50_ = 6.83 ± 0.76 mg/mL) and *C. sativum* (IC_50_ = 6.24 ± 0.86 mg/mL) EOs demonstrated similar antidiabetic activity, which is about nine-fold lower than the standard drug, acarbose (IC_50_ = 0.73 ± 0.10 mg/mL); however, the EO mixture (IC_50_ = 0.75 ± 0.15 mg/mL) displayed equipotent activity to acarbose. Our finding justifies the increased dietary intake of coriander seeds in decreasing the oxidative burden in DM. Terpenes have been provided for their antidiabetic potential. Studying the EO from *Harita cheirifolia* L., Majouli et al. [64] have reported that among the active monoterpenes, p-cymene and γ-terpinene revealed potent inhibitory effects. The strongest α-glucosidase inhibition effect was also displayed by *Sideritis galactic* EO containing high levels of β-pinene (32.2%), and the activity was ascribed to the high level of monoterpene hydrocarbons.

### 2.5. Pharmacokinetics Profiling of the Major Identified Components from C. carvi and C. sativum EOs

The pharmacokinetic profile of the major identified compounds defines their absorption, distribution, metabolism, excretion, and toxicity (ADMET) properties, which will be carefully considered in the early stages of drug development, leading to a significant reduction in the number of compounds that failed in clinical trials. The data depicted in Table 8 outlined that log BB values were in the range of 0.818 to 0.478 for all components, indicating their ability to penetrate the blood-brain barrier moderately and the fact that they will be highly distributed. They also manifested high Caco-2 permeability expressed by log Papp values > 0.90 cm/s. The volume of distribution (VDss) gives an indication about the circulation of a medicine at an equal level of blood plasma. Results showed that amongst the tested compounds, only α-pinene, β-pinene and linalool exhibited VDss values >0.45, suggesting that they may be more distributed in tissue rather than plasma. All tested compounds were predicted not to be a substrate or inhibitor of the human cytochrome P450 (CYP) isoforms (CYP2D6, CYP3A4, CYP1A2, CYP2C19, CYP2C9, CYP2D6 and CYP3A4), which are involved in drug metabolism. The transfer of a candidate compound by OCT2 gives useful information concerning its clearance, as well its potential contraindications. None of them are OCT2 substrates. The toxicity profile including AMES test, the human ether-a-go-go related gene (hERG), hepatotoxicity, and skin sensitization revealed that the selected identified compounds do not have any toxicity (Table 8).

Water solubility ≤ −4 is soluble; Intestinal absorption below 30% indicates poor absorbance; Blood brain barrier permeability ≤ −1 is considered poorly distributed to the brain; Central nervous system (CNS) permeability ≥ −2 is considered to penetrate the CNS; Low total clearance (logCLtot) value indicates high drug half lifetime.

**Figure 2:**
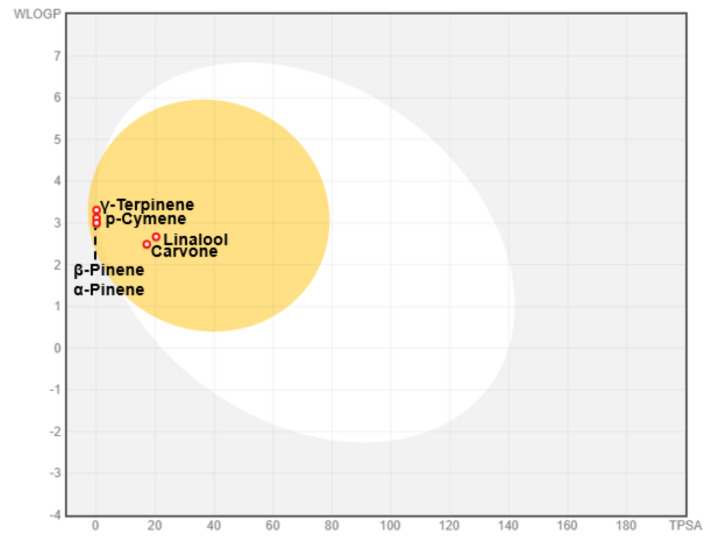
Boiled egg plot of WLogP vs. TPSA. Yellow indicates BBB-permeant, white indicates gastrointestinal permeant, blue indicates P-glycoprotein, and red indicates P-glycoprotein.

The BOILED-Egg graph (WLOGP vs. TPSA) prediction of GI absorption and BBB permeation, which helps in the computation of polarity and lipophilicity, revealed that all molecules fall in the yellow region with red points indicating that they possess a high probability of brain penetration and are non-substrate of P-gp (PGP-) (Figure 2).

## 3. Materials and Methods

### 3.1. Plant Material and Extraction of EOs

Our work focused on the seeds (achenes) of two spices: caraway and coriander. These two species were collected from a farmer in the Kelibia region, North-East of Tunisia (latitude 36,965918; longitude 11,037080) and identified according to the flora of Tunisia. The seeds were separated and then dried at room temperature for about ten days. Once dried, the plant material was extracted for EOs. An amount of 100 g of aerial part was transferred to hydro-distillation for 3 h with 500 mL distilled water using a Clevenger-type apparatus. The distilled EO was dried over anhydrous sodium sulfate, filtered, and stored at 4 °C. The yield was calculated based on the dried weight of the sample.

### 3.2. Essential Oils Analysis

#### 3.2.1. Gas Chromatography (GC)

A Hewlett-Packard 5890 series II gas chromatograph equipped with HP-5MS capillary column (30 m × 0.25 mm i.d., film thickness 0.25 µm; Hewlett-Packard, Palo Alto, CA, USA) and connected to a flame ionization detector (FID) was applied using the following conditions. The column temperature was programmed at 50 °C for 1 min, then increased by 7 °C/min to 250 °C, and then left at 250 °C for 5 min. The injection port temperature was 240 °C, and the detector temperature was 250 °C (split ratio: 1/60). The carrier gas was helium with a flow rate of 1.2 mL/min, and the analyzed sample volume was 2 µL. The percentages of the constituents were calculated by the electronic integration of FID peak areas, without the use of response factor correction. The mean percentage of compounds in the EO represents the average calculated from three individual experiments. Retention indices (RI) were calculated for separate compounds relative to C6-C28 *n*-alkanes mixture.

#### 3.2.2. Gas Chromatography-Mass Spectrometry (GC-MS)

The isolated volatile compounds were analyzed by GC-MS, using a Hewlett-Packard 5890 series II gas chromatograph. The fused HP-5MS capillary column (the same as that used in the GC analysis) was coupled to a HP 5972A masse-selective detector (Hewlett-Packard, Palo Alto, CA, USA). The oven temperature was programmed at 50 °C for 1 min, then increased by 7 °C/min to 250 °C, and then left at 250 °C for 5 min. The temperature of the injection port was set to 250 °C and that of the detector to 280 °C (split ratio: 1/100). The carrier gas was helium with a flow rate of 1.2 mL/min, and the analyzed sample volume was 2 µL. The mass spectrometer conditions were as follows: ionization voltage, 70 eV; ion source temperature, 150 °C; electron ionization mass spectra were acquired over the mass range 50–550 *m*/*z*. The components of these oils were identified following the same protocol as Hajlaoui et al. [27].

### 3.3. Antioxidant Activity

#### 3.3.1. Scavenging Ability on DPPH Radical

The DPPH quenching ability of the EO was measured according to the same experiment as described by Felhi et al. [65,66]. Anti-radical activity was expressed as IC_50_ (µg/mL) values, reflecting the extract doses required to cause a 50% inhibition. A lower IC_50_ value corresponded to a higher antioxidant activity of plant extract. 

#### 3.3.2. Superoxide Anion Radical-Scavenging Activity

Superoxide anion scavenging activity was assessed using the method described by Saini et al. [67] with slight modifications. The reaction mixture contained 0.2 mL of EO assayed at different concentrations, 0.2 mL of 60 mM PMS stock solution, 0.2 mL of 677 mM NADH, and 0.2 mL of 144 mM NBT, all in phosphate buffer (0.1 mol/L, pH 7.4). After incubation at ambient temperature for 5 min, absorbance was read at 560 nm against a blank. Antioxidant activity was evaluated based on IC_50_ values, which was defined as the amount of antioxidant needed to reduce the generation of superoxide radical anions by 50% and expressed as μg/mL (as determined from three replicates per treatment). The inhibition percentage of superoxide anion generation was calculated using the following formula:

Superoxide quenching (%) = [(A_0_ − A_1_) × 100]/A_0_

where, A_0_ and A_1_ had the same references presented in the above Equation.

#### 3.3.3. Reducing Power

The ability of the EO to reduce Fe^3+^ was assayed using the method described by Bakari et al. [68] and Kadri et al. [69] Briefly, 1 mL of the EO was mixed with 2.5 mL of phosphate buffer (0.2 M, pH 6.6) and 2.5 mL of 1% K_3_Fe(CN)_6_. Absorbance was measured at 700 nm. The mean of absorbance values was plotted against concentration values, and a linear regression analysis was performed. Increased absorbance of the reaction mixture indicated increased reducing power. The EC_50_ value (µg/mL) is the effective concentration at which absorbance was 0.5 for reducing power. BHT and ascorbic acid were used as positive control. 

#### 3.3.4. Chelating Effect on Ferrous Ions

The use of the ferrozine method was assessed to evaluate in vitro chelating power as reported by Ballester-Costa et al. [70]. The completion of the kinetics of this activity to determine the concentration that is 50% of chelating of ferric iron, the value of EC_50_ (µg.mL^−1^) corresponds to the lower efficiency of the oil highest.

#### 3.3.5. β-Carotene-Linoleic Acid Model System (β-CLAMS)

The β-CLAMS method is based on the discoloration of β-carotene by the peroxides generated during the oxidation of linoleic acid at an elevated temperature, and was performed based on the protocol done by Kadri et al. [69]. In this study the β-CLAMS was modified for the 96-well micro-plate reader. In brief, the β-carotene was dissolved in 2 mL of CHCl_3_, to which 20 mg of linoleic acid and 200 mg of tween 40 were added. The results are expressed as IC_50_ values (µg/mL). All samples were prepared and analyzed in triplicate.

### 3.4. Antimicrobial Activity

#### 3.4.1. Microorganisms

The microorganisms tested in this study belonged to 18 reference bacterial strains and 5 fungal strains, which are presented in Table 3 and Table 4, respectively. The bacterial species consisted of 6 Gram-positive and 12 Gram-negative bacterial strains. The fungal species belonged to four ATCC *Candida* strains and one *Saccharomyces* strain. A sterile cotton swab (Nippon Menbo, Tokyo, Japan) was immediately cultured into Sabouraud Chloramphenicol agar (Bio-Rad^®^, Mions, France) to obtain isolated colonies. 

#### 3.4.2. Disc-Diffusion Assay

Antimicrobial activity testing was performed according to the protocol described by Vuddhakul et al. [71] and slightly modified by Hajlaoui et al. [72] and Snoussi et al. [73] for *Vibrio* spp. strains. For the experiments, a loopful of the microorganisms working stocks were enriched on a tube containing 9 mL of Mueller-Hinton broth (for bacteria) and Sabouraud Chloramphenical broth (for Yeast strains), then incubated at 37 °C for 18 to 24 h. The overnight cultures were used for the antimicrobial activity of the EO used in this study, and optical density was adjusted at 0.5 McFarland turbidity standards with a DENSIMAT (BioMérieux^®^, Marcy l’Etoile, France). The inoculums of the respective bacteria and fungi were streaked onto MH or SB agar plates using a sterile swab. For *Vibrio* strains, the MH medium was supplemented with 1% NaCl. 

Sterile filter discs (diameter 6 mm, Whatman paper No. 3) were impregnated with 10 μL of EO placed on the appropriate agar media (SB, MH and MH + 1%NaCl). Gentamycin (10 μg/disc) and Amphotericin B (20 μg/disc) were used as positive reference standards to determine the sensitivity of one strain/isolate to each of the tested microbial species. Antibiotic susceptibility was determined using the Kirby–Bauer method on Mueller Hinton agar plates supplemented with 1% NaCl. After incubation at 37 °C for 18 to 24 h, the diameter of inhibition zone was measured with 1 mm flat rule, and diameters were interpreted according to the Committee of the French Society of the Antibiogram [74]. The dishes were incubated at 37 °C for 18–24 h for microbial strains. The diameter of inhibition zones around each of the discs was taken as a measure of antimicrobial activity. Each experiment was carried out in triplicate, and the mean diameter of the inhibition zone was recorded.

#### 3.4.3. Micro-Well Determination of MIC, MBC and MFC

Minimal inhibition concentration (MIC), minimal bactericidal concentration (MBC) and minimal fungicidal concentration (MFC) values were determined for all bacterial and fungal strains used in this study as described by Hajlaoui et al. [72]. A 100 μL aliquot from the stock solutions of EO was added into the first wells. Then, 100 μL from the serial dilutions were transferred into eleven consecutive wells. The last well containing 195 μL of nutrient broth without EO and 5 μL of the inoculum on each strip was used as the negative control. The final volume in each well was 200 μL. The plates were incubated at 37 °C for 18–24 h. The EO tested in this study was screened two times against each organism. The MIC value was defined as the lowest concentration of the compounds to inhibit the growth of the microorganisms. The MBC and MFC values were interpreted as the highest dilution (lowest concentration) of the sample, which showed clear fluid with no development of turbidity and without visible growth. MBC/MIC and MFC/MIC ratios were also calculated. All tests were performed once. 

### 3.5. Enzyme Inhibition Assays

#### 3.5.1. Anti-Acetylcholinesterase Inhibitory Assay

Acetylcholinesterase enzymatic activity was measured using a slightly modified version of the method described by Ingkaninan et al. [75]. In brief, 98 μL (50 mM) Tris–HCl buffer pH 8, 30 μL sample, and 7.5 μL acetylcholinesterase solution containing 0.26 U/mL were mixed in an ELISA well plate and left to incubate for 15 min. Subsequently, 22.5 μL of AChI (acetylthiocholine iodide) 0.023 mg/mL and 142 μL of (3 mM) DTNB were added. The absorbance at 405 nm was read when the reaction reached the equilibrium. A control reaction was carried out using water instead of extract. Galanthamine was used as a positive control. The absorbance value obtained was considered 100% activity. Inhibition (%) was calculated using the following equation:

I% = 100 − (A_sample_/A_control_) × 100

where, A_sample_ refers to the absorbance of the reaction containing the extract and A_control_ to the absorbance of the reaction control. Tests were carried out in triplicate, and a blank with Tris–HCl buffer instead of enzyme solution was performed. An extract concentration providing 50% inhibition (IC_50_) was obtained by plotting the inhibition percentage against extract solution concentrations.

#### 3.5.2. α-Glucosidase Inhibitory Assay 

The α-glucosidase assay of the tested EOs was conducted according to the standard method with slight modifications [75]. Inside the 96-well plate, 50 µL of phosphate buffer (100 mM, pH = 6.8), 10 µL α-glucosidase (1 U/mL), 20 µL of samples, and standard acarbose of different concentration were incubated for 15 min at 37 °C. Briefly, 20 µL of 5 mM substrate (4-nitrophenyl β-d-glucopyranoside) was added to each well and left to incubate for 20 min at 37 °C. The reacting mixture was stopped after incubation by adding 0.1 M sodium carbonate (50 µL). The release of p-nitrophenol of the reacting mixture relating to the activity of the enzyme was read at a wavelength of 405 nm using a multiplate reader (Multiskan, Thermo Scientific, Waltham, MA, USA). The enzyme inhibition rate, expressed as percentage of inhibition, was calculated using the following formula:

Percentage inhibitory activity (%) = (1 − A/B) × 100

where, A is the absorbance in the presence of test substance, and B is the absorbance in the presence of phosphate buffer (control). The results are expressed as IC_50_ values (µg/mL). All samples were prepared and analyzed in triplicate.

### 3.6. Pharmacokinetics Study

The pharmacokinetic and drug-likeness properties of the selected compounds were estimated using ADME (absorption, distribution, metabolism and excretion) descriptors by a SwissADME online server (http://www.swissadme.ch/, accessed on 20 April 2021) and pkCSM (http://biosig.unimelb.edu.au/pkcsm/prediction, accessed on 22 April 2021) online tools [76,77,78].

### 3.7. Statistical Analysis

All experiments were performed in triplicates, and average values were calculated using the SPSS 25.0 statistical package (version 25, Chicago, IL, USA) for Windows. Differences in means were calculated using the Duncan’s multiple-range tests for means with a 95% confidence interval (*p* ≤ 0.05).

## 4. Conclusions

As mentioned above, combined *C. carvi* and *C. sativum* seed EOs exhibited potent antioxidant effects and a broad spectrum of antimicrobial activity against the tested pathogenic strains, even higher than each EO alone. Also, the strongest inhibiting power of AChE and α-glucosidase enzymes have been outlined for the first time demonstrating the highest activity of the mixture, especially the antidiabetic one. The biological properties of this EO mixture, along with its pharmacokinetic profile, support the efficiency and traditional applications of these species in food, show that mixed EOs might provide an alternative way to fight microbial contamination, and indicate that EOs may be potential health-promoting antidiabetic and anti-Alzheimer agents.

## Figures and Tables

**Table 2:** DPPH test, superoxide anion radical-scavenging activity, reducing power, chelating power, and β-carotene of two EOs (*C. carvi* and *C. sativum*) and their mixture compared to authentic standards (BHT and EDTA).

	DPPH IC_50_ (μg.mL^−1^)	Superoxide Anion IC_50_ (μg.mL^−1^)	Reducing Power EC_50_ (μg.mL^−1^)	Chelating Power EC_50_ (μg.mL^−1^)	β-Carotene IC_50_ (μg.mL^−1^)
*C. sativum*	38.83 ± 0.76 ^a^	37.00 ± 1.73 ^a^	24.00 ± 1.53 ^a^	70.00 ± 0.81 ^a^	25.70 ± 1.02 ^a^
*C. carvi*	34.00 ± 3.46 ^b^	28.00 ± 7.00 ^b^	18.00 ± 1.00 ^b^	36.33 ± 4.10 ^b^	19.00 ± 2.16 ^b^
Mixture	19.00 ± 1.00 ^c^	10.33 ± 0.58 ^c^	11.33 ± 1.53 ^c^	31.33 ± 0.47 ^b^	11.16 ± 0.84 ^c^
BHT	11.5 ± 0.62 ^d^	1.60 ± 0.20 ^d^	23.00 ± 1.00 ^a^	-	4.60 ± 1.60 ^d^
EDTA	-	-	-	32.50 ± 1.32 ^b^	-

^a–d^: each value represents the average of 3 repetitions. Means (three replicates) followed by the same letter are not significantly different at *p* < 0.05.

**Table 3:** Inhibition zones of growth (IZ mm ± SD), showing the qualitative antibacterial activity of two EOs (*C. carvi* and *C. sativum*) and their mixture against human pathogenic bacteria compared to standard antibiotics (gentamycin and tetracycline).

		IZ (mm ± SD)		
Strains	*C. carvi*	*C. sativum*	Mixture (*v*/*v*)	Antibiotics
**Gram positive bacteria**				**Gentamycin**
*S. epidermidis* CIP 106510	16.33 ± 0.57 ^cC^	14.00 ± 1.00 ^deD^	18.66 ± 0.57 ^bcdB^	21.33 ± 0.58 ^fghA^
*S. aureus* ATCC 25923	15.66 ± 0.57 ^cdC^	11.66 ± 0.57 ^fD^	18.00 ± 0.00 ^bcdB^	32.67 ± 0.58 ^aA^
*M. luteus* NCIMB 8166	25.00 ± 0.00 ^aB^	21.66 ± 1.15 ^aC^	25.66 ± 0.57 ^aB^	27.67 ± 1.53 ^bA^
*E. feacalis* ATCC 29212	16.33 ± 0.57 ^cC^	12.33 ± 0.57 ^fD^	18.33 ± 0.57 ^bcdB^	26.00 ± 1.00 ^cA^
*B. cereus* ATCC 11778	18.33 ± 0.57 ^bC^	16.00 ± 1.00 ^cD^	20.66 ± 0.57 ^bcB^	26.00 ± 1.00 ^cA^
*B. cereus* ATCC 14579	19.00 ± 1.00 ^bC^	17.33 ± 0.57 ^bD^	22.33 ± 0.57 ^abB^	28.00 ± 0.00 ^bA^
**Gram negative bacteria**				
*E. coli* ATCC 35218	12.00 ± 1.00 ^ghC^	13.00 ± 0.00 ^efC^	14.66 ± 0.57 ^defB^	22.00 ±1.00 ^efgA^
*L. monocytogenes* ATCC19115	15.33 ± 0.57 ^cdC^	12.00 ± 0.00 ^fD^	18.00 ±0.00 ^efB^	23.00 ± 0.00 ^deA^
*P. aeruginosa* ATCC 27853	9.66 ± 1.15 ^iC^	8.33 ± 0.57 ^gC^	12.00 ± 1.00 ^fB^	17.00 ± 1.00 ^iA^
*S. typhimurium* LT2 DT104	11.00 ± 0.00 ^hC^	9.33 ± 1.15 ^gD^	14.33 ± 1.15 ^defB^	20.33 ± 0.57 ^hA^
***Vibrio* strains**				**Tetracycline**
*V. parahaemolyticus* ATCC17802	13.33 ± 0.57 ^efC^	12.33 ± 0.57 ^fC^	16.66 ± 0.57 ^cdeB^	18.33 ± 0.57 ^iA^
*V. alginolyticus* ATCC 33787	14.66 ± 0.57 ^deB^	12.00 ± 1.00 ^fC^	15.66 ± 0.57 ^defB^	20.67 ± 0.57 ^ghA^
*V. proteolyticus* ATCC15338	11.00 ± 1.00 ^hC^	9.66 ± 1.54 ^gC^	13.00 ± 1.54 ^efB^	18.00 ± 1.00 ^iA^
*V. furnisii* ATCC 35016	14.33 ± 0.57 ^deC^	12.66 ± 0.57 ^efD^	16.33 ± 0.57 ^cdefB^	22.67 ± 0.57 ^defA^
*V. mimicus* ATCC 33653	12.33 ± 0.57 ^fgC^	9.66 ± 0.57 ^gD^	15.33 ± 0.57 ^defB^	21.00 ± 0.00 ^ghA^
*V. natrigens* ATCC 14048	13.33 ± 0.57 ^efC^	12.00 ± 1.00 ^fD^	11.33 ± 9.81 ^cdeB^	23.67 ± 0.57 ^dA^
*V. carhiaccae* ATCC 35084	14.66 ± 1.15 ^deC^	13.00 ± 1.00 ^efD^	18.33 ± 0.57 ^bcdB^	23.33 ± 0.58 ^deA^
*V. fluvialis* ATCC 33809	14.33 ± 0.57 ^deC^	14.66 ± 0.57 ^dC^	18.66 ± 0.57 ^bcdB^	23.67 ± 0.57 ^dA^

SD = standard deviation; IZ = inhibition zone diameter (mm) around the discs (6 mm) impregnated with 10 μL of essential oil and 10 μg/disc for gentamycin and tetracycline; ^a–i, A–D^ = each value represents the average of 3 repetitions. Means followed by the same letters are not significantly different at *p* = 0.05 based on Duncan’s multiple range test. Lower case letters are used to compare EO means with different strains, while capital letters are used to compare means between EOs for the same strain.

**Table 4:** Inhibition zones of growth (IZ mm ± SD), showing the qualitative antifungal activity of two EOs (*C. carvi* and *C. sativum*) and their mixture against human pathogenic fungal strains compared to a standard antifungal (Amphotericin B).

IZ (mm ± SD)				
Strains	*C. carvi* EO	*C. sativum* EO	Mixture EO (*v*/*v*)	Amphotericin B
*C. albicans* *ATCC 90028*	15.33 ± 0.57 ^abB^	12.33 ± 0.57 ^abC^	18.00 ± 0.00 ^aA^	18.00 ± 0.00 ^aA^
*C. glabrata* *ATCC 90030*	14.66 ± 1.15 ^bB^	13.33 ± 0.57 ^aB^	17.66 ± 0.57 ^aA^	16.33 ± 0.57 ^bA^
*C. parapsilosis ATCC* *22019*	14.00 ± 1.00 ^bC^	11.66 ± 0.57 ^bD^	15.66 ± 1.15 ^bB^	17.33 ± 0.57 ^aA^
*C. krusei* *ATCC 6258*	16.66 ± 1.15 ^aA^	12.00 ± 0.00 ^bB^	17.00 ± 1.00 ^abA^	16.00 ± 0.00 ^bA^
*S. cerevisae*	13.66 ± 0.57 ^bC^	12.00 ± 1.00 ^bD^	16.00 ± 0.00 ^bB^	18.00 ± 0.00 ^aA^

SD = standard deviation; IZ = inhibition zone diameter (mm) around the discs (6 mm) impregnated with 10 μL of essential oil and 20 μg/disc for Amphotericin B; ^a,b, A–D^ = each value represents the average of 3 repetitions. Means followed by the same letters are not significantly different at *p* = 0.05 based on Duncan’s multiple range test. Lower case letters are used to compare EO means with different strains, while capital letters are used to compare means between EOs for the same strain.

**Table 5:** Minimal inhibition concentration (MIC), minimal bactericidal concentration (MBC), and MBC/MIC ratio showing quantitative antibacterial activity of two EOs (*C. carvi* and *C. sativum*) and their mixture against human pathogenic bacteria compared to standard antibiotics (gentamycin and tetracycline).

Microorganisms Tested	*C. carvi* EO			*C. sativum* EO			Mixture (*v*/*v*) EOs			Antibiotics		
	MIC mg/mL	MBC mg/mL	MBC/MIC (Interpretation)	MIC mg/mL	MBC mg/mL	MBC/MIC (Interpretation)	MIC mg/mL	MBC mg/mL	MBC/MIC (Interpretation)	MIC mg/mL	MBC mg/mL	MBC/MIC
*S. epidermidis* CIP 106510	0.117	0.469	4 (Bactericidal)	0.469	1.875	4 (Bactericidal)	0.029	0.117	4 (Bactericidal)	0.009	0.039	4
*S. aureus* ATCC 25923	0.117	0.469	4 (Bactericidal)	0.234	0.938	4 (Bactericidal)	0.059	0.234	4 (Bactericidal)	0.004	0.019	4
*M. luteus* NCIMB 8166	0.059	0.234	4 (Bactericidal)	0.234	0.938	4 (Bactericidal)	0.015	0.059	4 (Bactericidal)	0.004	0.019	4
*E. feacalis* ATCC 29212	0.059	0.469	8 (Bacteriostatic)	0.469	0.938	2 (Bactericidal)	0.117	0.234	2 (Bactericidal)	0.004	0.019	4
*B. cereus* ATCC 11778	0.117	0.469	4 (Bactericidal)	0.938	1.875	2 (Bactericidal)	0.059	0.117	2 (Bactericidal)	0.004	0.039	8
*B. cereus* ATCC 14579	0.117	0.469	4 (Bactericidal)	0.938	1.875	2 (Bactericidal)	0.059	0.234	4 (Bactericidal)	0.004	0.039	4
*E. coli* ATCC 35218	0.469	1.875	4 (Bactericidal)	0.938	3.750	4 (Bactericidal)	0.117	0.469	4 (Bactericidal)	0.009	0.039	4
*L. monocytogenes* ATCC19115	0.469	1.875	4 (Bactericidal)	0.938	1.875	2 (Bactericidal)	0.059	0.234	4 (Bactericidal)	0.019	0.078	4
*P. aeruginosa* ATCC 27853	1.875	3.750	2 (Bactericidal)	1.875	7.500	4 (Bactericidal)	0.469	0.938	2 (Bactericidal)	0.019	0.15	4
*S. typhimurium* LT2 DT104	0.234	0.938	4 (Bactericidal)	0.938	1.875	2 (Bactericidal)	0.059	0.117	2 (Bactericidal)	0.019	0.039	2
*V. parahaemolyticus* ATCC17802	0.938	3.750	4 (Bactericidal)	0.234	1.875	8 (Bacteriostatic)	0.117	0.234	2 (Bactericidal)	0.039	0.078	2
*V. alginolyticus* ATCC 33787	0.234	1.875	8 (Bacteriostatic)	1.875	7.500	4 (Bactericidal)	0.059	0.469	8 (Bacteriostatic)	0.019	0.078	4
*V. proteolyticus* ATCC15338	0.469	1.875	4 (Bactericidal)	1.875	3.750	2 (Bactericidal)	0.117	0.469	4 (Bactericidal)	0.009	0.039	4
*V. furnisii* ATCC 35016	0.469	3.750	8 (Bacteriostatic)	1.875	7.500	4 (Bactericidal)	0.117	0.469	4 (Bactericidal)	0.009	0.039	4
*V. mimicus* ATCC 33653	0.938	3.750	4 (Bactericidal)	1.875	7.500	4 (Bactericidal)	0.234	0.938	4 (Bactericidal)	0.039	0.078	2
*V. natrigens* ATCC 14048	1.875	3.750	2 (Bactericidal)	1.875	3.750	2 (Bactericidal)	0.469	0.938	2 (Bactericidal)	0.019	0.039	2
*V. carhiaccae* ATCC 35084	0.938	1.875	2 (Bactericidal)	1.875	7.500	4 (Bactericidal)	0.117	0.234	2 (Bactericidal)	0.039	0.078	2
*V. fluvialis* ATCC 33809	0.469	1.875	4 (Bactericidal)	0.469	3.75	8 (Bacteriostatic)	0.117	0.469	4 (Bactericidal)	0.019	0.039	2

**Table 6:** Minimal inhibition concentration (MIC), minimal fungicidal concentration (MFC) and ratio MFC/MIC showing antifungal activity for two EOs (*C. carvi* and *C. sativum*) and their mixture against human pathogenic fungi compared to standard antifungals (Amphotericin B). MIC and MBC values are expressed in mg/mL.

	*C. carvi* EO			*C. sativum* EO			Mixture EO (*v*/*v*)			Amphotericin B		
Strains	CMI	CMF	CMF/CMI (Interpretation)	CMI	CMF	CMF/CMI (Interpretation)	CMI	CMF	CMF/CMI (Interpretation)	CMI	CMF	CMF/CMI (Interpretation)
*C. albicans ATCC* *90028*	0.059	0.469	8 (Fungistatic)	0.469	1.875	4 (Fungicidal)	0.029	0.117	4 (Fungicidal)	0.078	0.31	4 (Fungicidal)
*C. glabrata* *ATCC 90030*	0.059	0.234	4 (Fungicidal)	0.234	0.938	4 (Fungicidal)	0.029	0.117	4 (Fungicidal)	0.009	0.078	9 (Fungistatic)
*C. parapsilosis ATCC* *22019*	0.059	0.234	4 (Fungicidal)	0.469	0.938	2 (Fungicidal)	0.029	0.117	4 (Fungicidal)	0.039	0.078	2 (Fungicidal)
*C. krusei* *ATCC 6258*	0.059	0.469	8 (Fungistatic)	0.234	1.875	8 (Fungistatic)	0.029	0.117	4 (Fungicidal)	0.009	0.019	2 (Fungicidal)
*S. cerevisae*	0.029	0.234	8 (Fungistatic)	0.234	0.938	4 (Fungicidal)	0.015	0.117	8 (Fungistatic)	0.009	0.039	4 (Fungicidal)

**Table 7:** Inhibitory activities of acetylcholinesterase and α-glucosidase of two EOs (*C. carvi* and *C. sativum*) and their mixture compared to authentic standards (Galanthamine, Acarbose).

IC_50_ (mg/mL)		
	Acetylcholinesterase	α-Glucosidase
*C. sativum* EO	0.68 ± 0.03 ^c^	6.24 ± 0.86 ^a^
*C. carvi* EO	0.82 ± 0.05 ^b^	6.83 ± 0.76 ^a^
Mixture	0.63 ± 0.02 ^c^	0.75 ± 0.15 ^b^
Acarbose	-	0.73 ± 0.10 ^b^
Galanthamine	1.05 ± 0.05 ^a^	-

^a–c^: each value represents the average of 3 repetitions. Means (three replicates) followed by the same letter are not significantly different at *p* < 0.05.

**Table 8:** Pharmacokinetics profiles of the major identified components.

	γ-Terpinene	β-Pinene	α-Pinene	*p*-Cymene	Carvone	Linalool
**Absorption**						
Water solubility	−3.941	−4.191	−4.081	−2.324	−2.612	−3.733
Caco_2_ permeability	1.414	1.385	1.527	1.413	1.493	1.38
Intestinal absorption	96.219	95.525	93.544	97.702	93.163	96.041
Skin permeability	−1.489	−1.653	−1.192	−2.145	−1.737	−1.827
P-g substrate	No	No	No	No	No	No
P-g I/II inhibitor	No	No	No	No	No	No
**Distribution**						
VDss (human)	0.412	0.685	0.697	0.179	0.152	0.667
Fraction unbound	0.42	0.35	0.159	0.53	0.484	0.425
BBB permeability	0.754	0.818	0.478	0.588	0.598	0.791
CNS permeability	−2.049	−1.857	−1.397	−2.478	−2.339	−2.201
**Metabolism**						
CYP2D6 substrate	No	No	No	No	No	No
CYP3A4 substrate	No	No	No	No	No	No
CYP1A2 inhibitor	No	No	Yes	No	No	No
CYP2C19 inhibitor	No	No	No	No	No	No
CYP2C9 inhibitor	No	No	No	No	No	No
CYP2D6 inhibitor	No	No	No	No	No	No
CYP3A4 inhibitor	No	No	No	No	No	No
**Excretion**						
Total Clearance	0.217	0.03	0.239	0.225	0.446	0.043
Renal OCT2 substrate	No	No	No	No	No	No
**Toxicity**						
AMES toxicity	No	No	No	No	No	No
hERG I/II inhibitors	No	No	No	No	No	No
Skin sensitization	No	No	Yes	Yes	No	Yes
Hepatotoxicity	No	No	No	No	No	No

## Data Availability

All data generated or analyzed during this study are included in this article.

## Sample Availability

The essential oils of *Carum carvi* L. and *Coriandrum sativum* L. are available from the authors.
